# Auranofin Inhibition of Thioredoxin Reductase in a Preclinical Model of Small Cell Lung Cancer

**DOI:** 10.1101/2023.05.07.539772

**Published:** 2023-05-09

**Authors:** Spenser S. Johnson, Dijie Liu, Jordan T. Ewald, Claudia Robles-Planells, Khaliunaa Bayanbold, Brian R. Wels, Shane R. Solst, M. Sue O’Dorisio, Bryan G. Allen, Yusuf Menda, Douglas R. Spitz, Melissa A. Fath

**Affiliations:** 1 The University of Iowa

## Abstract

Thioredoxin Reductase (TrxR) is a key enzyme in reactive oxygen species (ROS) detoxification and in redox regulation. Because cancer cells produce increased steady-state levels of ROS (i.e., superoxide and hydrogen peroxide), TrxR is viable target in clinical trials using the anti-rheumatic drug, auranofin (AF). To extend these observations to small cell lung cancer (SCLC), AF-mediated TrxR inhibition as well as tolerability and tumor growth inhibition was determined in a xenograft model. AF was administered intraperitoneal, daily or twice daily for 1 to 5 days in mice bearing DMS273 xenografts. AF uptake was determined by mass spectrometry of gold and inhibition of TrxR in the tumor was determined. The optimal dose was 4 mg/kg once daily resulting in 18 μM gold in the plasma and 50% inhibition of TrxR activity in DMS273 SCLC tumors. This regimen given for 14 days provided a trend for prolonged median survival from 17.5 to 22 days (p=0.058, N=20 controls, 19 AF) without causing changes in bodyweight, bone marrow toxicity, blood urea nitrogen or creatinine. These results support the hypothesis that AF is an effective inhibitor of TrxR and suggest that AF could be used as an adjuvant in radio-chemotherapy protocols to enhance therapeutic efficacy.

## Introduction

Lung cancer is the second most common cancer and small cell lung cancer (SCLC) makes up about 15% of lung cancer diagnoses. SCLC is a high-grade neuroendocrine tumor with characteristics of rapid tumor growth and high vascularity; two-thirds of patients have distant metastatic disease at initial diagnosis. Standard of care therapies for limited stage disease include cisplatin or carboplatin in combination with etoposide and thoracic irradiation. Improved outcomes with immunotherapy as concurrent primary or adjuvant therapy has been demonstrated but these combination therapies are still considered experimental.^1 2^ The median survival duration remains less than two years for patients with limited-stage disease and less than one year for patients with metastatic disease.^3^ Therefore, improved treatment options are of utmost importance for SCLC.

A hallmark of cancer is the increased steady-state levels of reactive oxygen species (ROS) often stemming from one electron reductions of O_2_ in mitochondrial electron transport chains to form superoxide radical (O_2_^·−^) which is rapidly converted to H_2_O_2_^4^. Cancer cells adapt to increased levels of ROS by upregulating endogenous antioxidant systems, including the scavenging enzymes superoxide dismutase and catalase, as well as enzymes in the glutathione-dependent and thioredoxin-dependent hydroperoxide metabolic pathways. The glutathione and thioredoxin systems play key roles in the overall cellular oxidation state because of their intracellular dithiol recycling nature.^5^ In particular, the thioredoxin system consists of the redox-active proteins thioredoxin (Trx) and the NADPH electron acceptor thioredoxin reductase (TrxR) which primarily function to reduce peroxiredoxin (Prx) during the catalytic reduction of hydroperoxides. TrxR has three known mammalian isoforms that can be found in the cytosol mitochondria, and one specific to spermatozoa.^6^ According to the Project Score database, knock-out of TrxR has shown an anti-tumoral effect in 21% of 324 cell-lines subjected to CRISPR-Cas9 screens ^7^ clearly indicating TrxR as a potential target for cancer treatment.

Originally developed in the 1980s for rheumatoid arthritis, auranofin (AF) was thought to exert it effects by inhibiting humoral immunity.^8^ The most common adverse reactions associated with AF treatment were gastrointestinal-as loose stools or diarrhea occurred in 39% of patients. Rashes and pruritis were also common, occurring in >10% of patients.^8 9,10^ In addition, gold salts caused thrombocytopenia in 0.7% of patients.^11^ Although diarrhea and stomach upset occurred in the initial month of treatment, other adverse events typically occurred only after prolonged therapy.^12^ Clinical AF use has declined due to the availability of more effective and better tolerated biologic treatments for rheumatoid arthritis.

AF, a gold phosphine compound, was recently discovered to irreversibly inhibit both cytosolic and mitochondrial TrxR.^13,14^ This discovery has led to the active investigation of repurposing AF as an anticancer agent. Currently, four clinical trials using AF are underway for chronic lymphocytic leukemia (NCT01419691, NCT01747798), ovarian cancer (NCT03456700), and recurrent lung cancer (NCT01737502). We have previously shown that treatment of breast cancer cells with AF before external beam radiation decreases the number of invading metastatic breast cancer cells as well as the number of breast cancer stem cells.^15^ We also demonstrated the suppressing TrxR with AF can sensitize breast cancer stem cells to ROS induced differentiation and cytotoxicity.^16^

The current study developed a SCLC xenograft model in female athymic nude mice and determined a safe and efficacious dose of AF that significantly inhibited TrxR in tumors. Complete blood counts, bone marrow hematopoietic stem cell and progenitor cell populations, renal and liver function evaluation by serum chemistry analyses, weight loss, and animal body conditioning as well as survival were examined after two-week dosing schedule of AF. These results provide a rigorous characterization of this SCLC xenograft model for testing the FDA approved thioredoxin reductase inhibitor, AF, as an adjuvant to radiation therapy in a preclinical setting.

## Methods and Materials

### Cell Culture

DMS273 human small cell lung cancer cells were obtained from American Type Culture Collection (ATCC) and maintained in RPMI 1640 media (Mediatech Inc., Manassas, VA) with 10% fetal bovine serum (Atlanta Biologicals, Lawrenceville, GA). Cultures were maintained in 5% CO_2_ and humidified in a 37°C incubator. Cells were used within 20 passages from ATCC and tested as mycoplasma negative.

### Animal Experiments

Female 6–8-week-old athymic-nu/nu nude mice were purchased from Envigo. Mice were housed in a pathogen-free barrier room in the Animal Care Facility at the University of Iowa and handled using aseptic procedures. All procedures were approved by the IACUC committee of the University of Iowa and conformed to the guidelines established by NIH. Mice were allowed at least one week to acclimate prior to beginning experimentation; food and water were made freely available. Tumor cells were inoculated into nude mice by subcutaneous injection of 0.1 mL aliquots of saline containing 1 × 10^6^ DMS273 cells into the right and/or left flank using 25-gauge needles. When tumor volumes measured approximately 100 mm^3^ treatments were started. Mouse health was monitored daily during drug treatment. Mice were terminally anesthetized using 200 μl intraperitoneal injection of ketamine/xylazine 17.5/2.5 μg/μl mix. When analgesia was achieved, as determined by lack of toe pinch response, blood was withdrawn via cardiac puncture followed by cervical dislocation.

### In vivo AF Dose Escalation

In the dose escalation study, five groups of three mice/group were given AF intraperitoneal (IP) once (QD) or twice (BID) a day for 5 days. AF was made by dissolving 5.0 mg AF in 250 μl of absolute ethanol in a 10 ml glass beaker. When completely dissolved 250 μl Kolliphor EL (Sigma C5135) and 4 ml of 0.9% normal saline for injection (Hospira) were added and pH was adjusted to 7–8 range using approximately 25 μl 8.4% sodium bicarbonate for injection (Hospira) followed by addition of normal saline for injection to adjust final volume to 8.33 ml and filtered using 200-micron filter into a sterile vial. The final AF concentration was 0.6 mg/ml and it was made fresh daily. Mice in the control group were administered vehicle without drug. Mice were euthanized 21 hours after the last QD dose and 9 hours after the last BID dose; tumors and organs were frozen in liquid nitrogen for later evaluation for TrxR activity. Terminal blood was collected via cardiac puncture under anesthesia and plasma separated via centrifugation at 10,000 rcf for 10 min.

### Biochemical Assays

Glutathione peroxidase 1 (GPx1) activity was determined spectrophotometrically using the method of Lawrence and Burk.^15^ Enzymatic activity was determined by measuring the disappearance of NADPH at 340 nm in the presence of GPx1 from cell pellets or standards. One unit of activity was defined as 1 μmole NAPDH oxidized/min at specified glutathione (GSH) concentration. Protein concentrations were determined by the Lowry assay.

TrxR activity was determined spectrophotometrically using the reduction of 5,5’-dithiobis(2-nitrobenzoic) acid (DTNB) with NADPH to 5-thio-2-nitrobenzoic acid (TNB), which generates a yellow color at 412 nm. (Millipore-Sigma CS0170). Enzymatic activity was determined by subtracting the time dependent increase in absorbance at 412 nm in the presence of the TrxR activity inhibitor, aurothioglucose from total activity. One unit of activity was defined as 1 μM TNB formed/(min·mg protein). Protein concentrations were determined by the Lowry assay.

### Determining Gold content in Plasma

Plasma samples were prepared by treatment with aqua regia followed by dilution and analysis using inductively coupled plasma mass spectrometry (ICPMS). Aqua regia was prepared from Optima grade (Fisher Scientific) hydrochloric and nitric acids (3:1, respectively). A 100-μL aliquot of plasma was transferred to a 15-mL centrifuge tube, 500 μL aqua regia added and the mixture heated on a hot block at 90 +/− 4 °C for 30 minutes. After cooling, volume was brought to 2.0 mL using deionized water. A 500-μL aliquot of the digestate was mixed with an equal volume of internal standard (10 μg/L Iridium) and analyzed using ICPMS (Agilent 7700) with an external calibration curve.

### Tumor growth and survival

In the survival experiments, 2 groups of mice (9–10/group) were administered AF at 4 mg/kg or vehicle interperitoneally once a day for 14 consecutive days. Mice were monitored and tumors measured daily using Vernier calipers (Vol = (Length × Width^2^)/2) and euthanized on the day when tumor size exceeded 1000 mm^3^. No mice died before reaching this endpoint. This experiment was repeated two times. Three mice in each group were euthanized 24 hours after the last dose of AF, and tumors were frozen at −80 °C for TrxR activity assay. Blood was collected via cardiac puncture under, see above, and plasma separated via centrifugation at 2500 rcf for 10 min and sent to Antech Diagnostics for clinical chemistry evaluation. Additional blood was obtained via mandibular vein and diluted 1:10 in PBS and a complete blood cell count (CBC) processed in a Siemens ADVIA 120 Hematology Analyzer.

### Bone marrow harvest and flow cytometry

Immediately after death, whole bone marrow cells were obtained by flushing one femur with PBS 2% FBS followed by erythrocyte elimination with ACK Lysing Buffer (10–548E, BioWhittaker, Lonza). The remaining cells were filtered through 40 μm pore size cell strainer and counted. 1×10^6^ cells were stained with Zombie Aqua (423102, BioLegend) for 20 min at room temperature for live/dead cells discrimination, followed by a 30 min surface staining on ice using PBS 1% BSA as staining buffer with allophycocyanin (APC)-conjugated, lineage negative (Lin−) cocktail (BD Bioscience), PerCP/Cy5.5-conjugated anti-Sca1 (Clone D7, BioLegend), APC/Cy7-conjugated anti-cKit (Clone 2B8, BioLegend), PE-conjugated anti-CD135 (Clone A2F10, BioLegend), FITC-conjugated anti-CD48 (Clone HM48–1, BioLegend), PE/Cy7-conjugated anti-CD150 (Clone TC15–12F12.2, BioLegend) and BV421-conjugated anti-CXCR4 (Clone L276F12, BioLegend). Cells were immediately analyzed using an LSR II flow cytometer (Becton Dickinson). The biomarkers were applied to identify multipotent hematopoietic stem cells (HSC) (Lin− cKit+ Sca1+, LSK), ST-HSC (short-term hematopoietic stem cells, Lin− cKit+ Sca1+ CD135− CD48− CD150−), LT-HSC (long-term hematopoietic stem cells, Lin− cKit+ Sca1+ CD135− CD48+CD150−), and committed progenitors (hematopoietic stems and progenitor cells, HSPC, Lin− cKit+ Sca1+ CD135−) using FlowJo Software (Treestar, Ashland, OR).

### Statistical analysis

The biochemical data analysis was performed using ANOVA multiple comparisons with uncorrected Fishers LSD on Graph Pad Prism software. The *in vivo* analysis regression analysis was used to model tumor growth as a nonlinear function of follow-up time and to make treatment group comparisons of estimated tumor means. Survival graphs were obtained with the methods of Kaplan–Meier and compared with log-rank tests.

## Results

### Dosing AF to a Maximal Effective Concentration in mice

**Figure 1 -:**
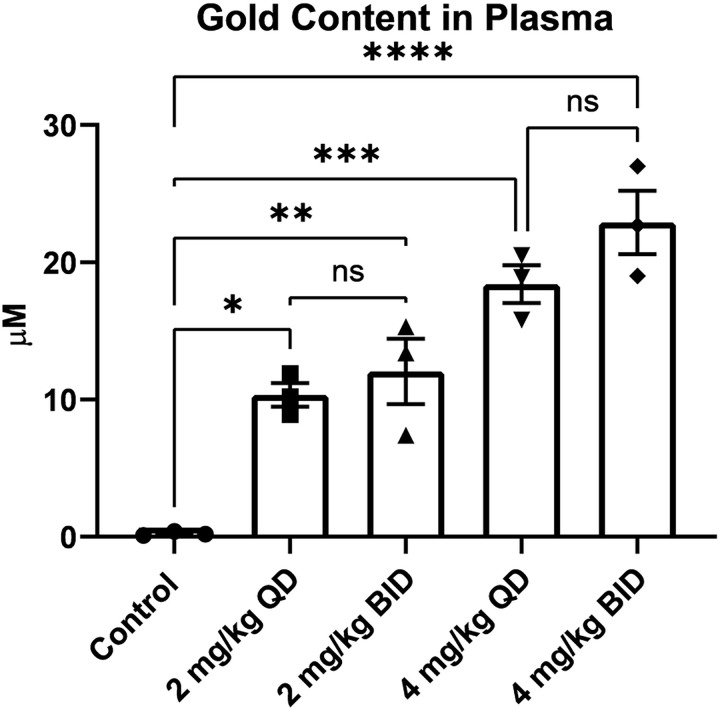
Gold circulates through blood plasma after IP injections. Mice were injected IP with AF for five days. Plasma samples were collected 9 hours after final BID dose and 21 hours after final QD dose. Gold was determined using inductively coupled plasma mass spectrometry ** = p-value < 0.01, **** = p-value < 0.0001. Analyzed by One-Way ANOVA multiple comparison.

**Figure 2 –:**
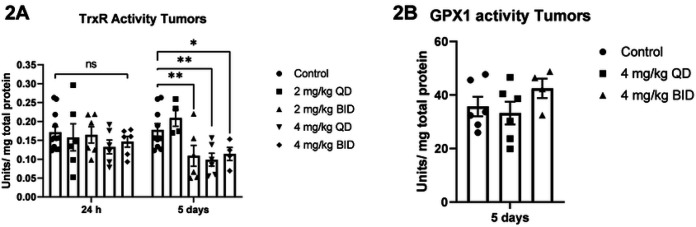
Auranofin selectively decreases TrxR activity in SCLC xenografts. Female athymic nude mice were xenografted with 5 ×10^6^ DMS273 cells and given AF IP injections after 5 days either QD or BID. Kidney, liver, and tumor samples collected for TrxR activity; results are plotted after 24 hours or 5 days of treatment. * = p-value < 0.05, ** = p-value < 0.01, *** = p-value < 0.001. Analyzed by Mix Effects ANOVA with Fishers LSD comparisons.

**Figure 3 –:**
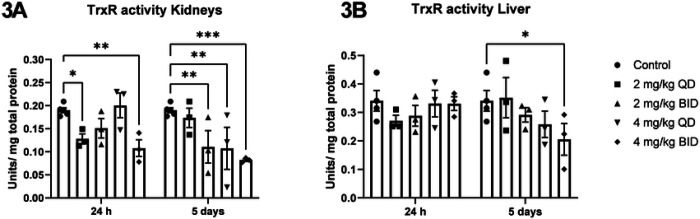
4mg/kg QD doses of AF results in a decrease in TrxR activity in mouse kidneys but not liver. Female athymic nude mice were xenografted with 5 ×10^6^ DMS273 cells and given AF IP injections for 5 days either once daily (QD) or twice daily (BID). Kidney, liver, and tumor samples were collected at euthanasia and evaluated for TrxR activity; results are plotted after 24 hours or 5 days of treatment. * = p-value < 0.05, ** = p-value < 0.01, *** = p-value < 0.001. Analyzed by two-way ANOVA with Fishers LSD comparisons.

Initially, we sought to determine a safe and effective dose of AF in mice with SCLC cell line DMS273 xenografts. AF (2 or 4 mg/kg) was administered IP once (QD) or twice (BID) daily up to 5 days. Plasma gold levels were measured nine hours after the last BID dosing or 21 hours after the last QD dosing. There was a significant increase in plasma gold content of all mice treated with AF (Figure 1). As expected, 4 mg/kg dosing resulted in significantly more gold in the plasma than 2 mg/kg dosing. There was not a significant difference between QD and BID dosing indicating that steady state levels of gold in the plasma can be maintained with five days of QD dosing as expected with the long half-life of AF reported in humans and rodents (Figure 1).^8,17^ To investigate if these plasma levels are effective at reducing TrxR activity we measured the activity in the DMS273 tumors. After 24 hours, AF had no effects on TrxR activity in the tumors, but by 5 consecutive days of dosing TrxR activity was inhibited by 50% at 2 mg/kg BID, 4 mg/kg QD, and 4 mg/kg BID (Figure 2A). Gpx1 is also a selenium-containing enzyme with an active thiol moiety, and it has been reported that high doses of AF can inhibit its activity.^18^ As a control for AF specificity for inhibition of TrxR, GPx1 activity was measured in the tumors after five days in the 4 mg/kg groups; there was no change in GPx1 activity (Figure 2B). TrxR activity in kidneys was significantly reduced by 2 mg/kg QD and 4 mg/kg BID at 24 hours. TrxR activity in kidneys was significantly inhibited by 5 consecutive days of dosing by 50% in 2 mg/kg BID, 4 mg/kg QD, and 4 mg/kg BID (Figure 3A). TrxR inhibition in liver was only observed at an AF dose of 4 mg/kg BID at five days (Figure 3B). No significant changes in weight or general health in any mice were observed (data not shown). Together these results suggest that 4 mg/kg QD is the optimal AF dose to achieve inhibition of TrxR activity in DMS273 tumors. Importantly, this dose also inhibited TrxR activity in the kidneys indicating potential nephrotoxicity.

### AF Effects on Tumor Growth, Survival, and Normal Tissue Injury

**Figure 4 –:**
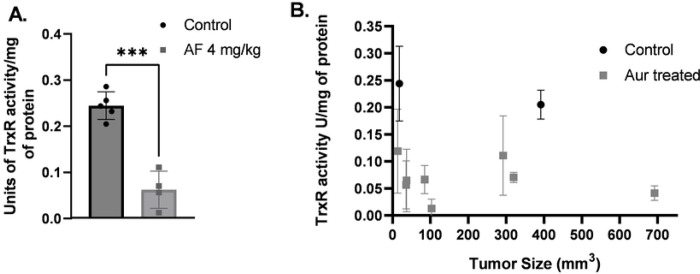
AF decreases TrxR activity in DMS273 tumors in vivo independent of tumor size. Small cell lung cancer (DMS273) xenografts were treated with Aur 4 mg/kg QD for 12 days then euthanized and evaluated for TrxR enzyme activity assays and results are plotted in aggregate (A) or as a function of tumor size at the end of treatment (B). * p<0.05 compared to untreated cells two-ways ANOVA with Fishers LSD comparisons.

**Figure 5 –:**
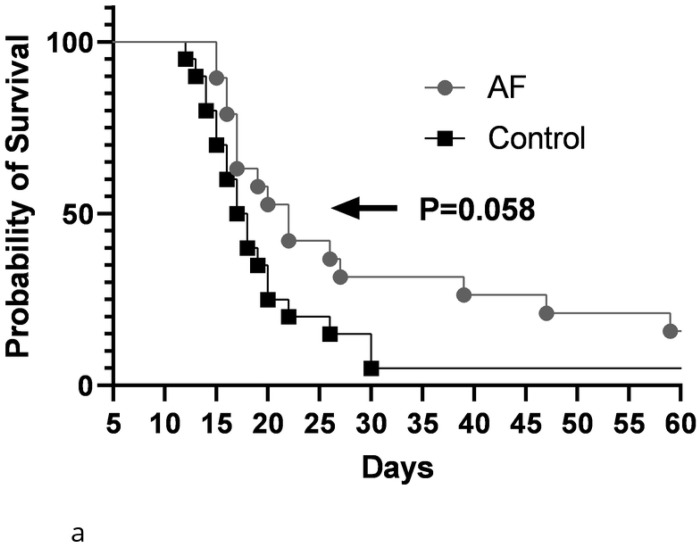

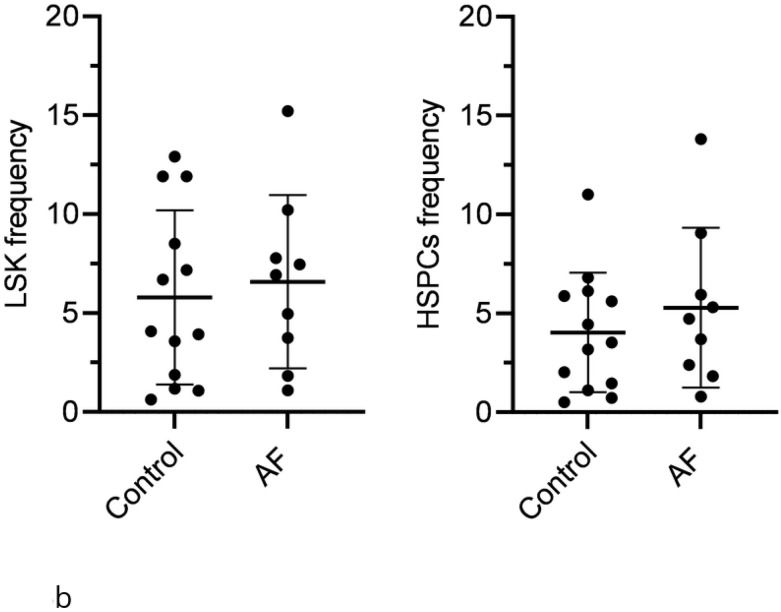

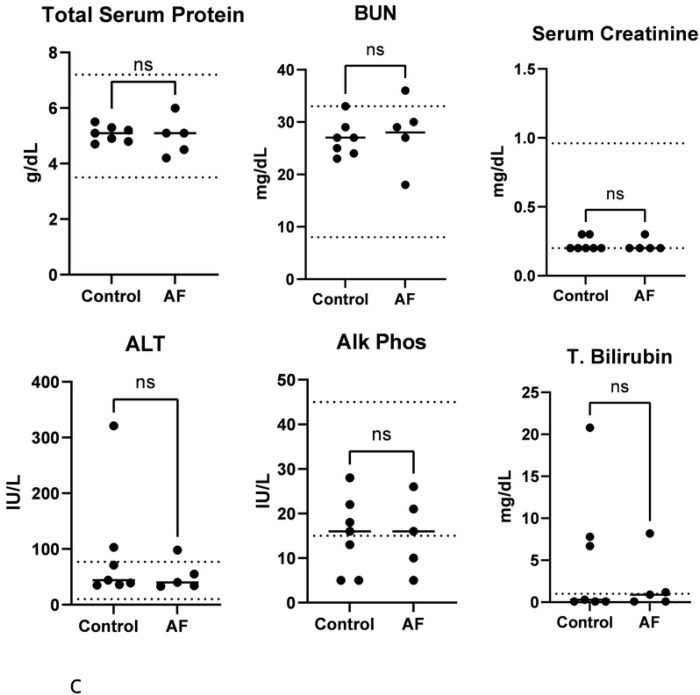
AF increases survival in DMS273 xenograft mice without causing toxcity. Female nude mice bearing DMS273 xenografts were treated with either vehicle or 4 mg/kg AF I.P every day for 14 days. Mice were euthanized when tumors were ≥ 1000 mm^3^. **A)** Kaplan-Meier curves were used to estimate survival. Log-rank (Mantel Cox test) for significance. **B)** Bone marrow was harvested from the femur at euthanasia and subjected to flow cytometry for HSCs (Lineage (Lin)− Sca-1+ c-Kit+, LSK) and HSPCs (Lin− cKit+ Sca1+ CD135−) populations. **C)** blood was draw via cardiac puncture at euthanasia and evaluate for clinical chemistry.

DMS273 cells were implanted subcutaneously into the right flank of nude female mice and allowed to grow for 6 days followed by treatment with AF at 4 mg/kg or vehicle control for 14 consecutive days. Tumor volumes and mice weights were measured every other day or daily as the tumor size progressed. Twenty-four hours after the last AF dose, 4 mice were euthanized, and their tumors collected to determine TrxR activity. AF significantly inhibited TrxR activity in the tumors by 75%, and this inhibition was independent of tumor volume (Figure 4A & B) indicating excellent penetration of the drug even in large tumors. When the tumors reached 1000 mm^3^ the mice were euthanized. The experiment was repeated in a second cohort of mice in an identical fashion. Kaplan Meier survival curves combining both cohorts resulted in an encouraging trend toward increased survival (median overall survival of 18 days in control versus 22 days in drug treated animals; p=0.058) (Figure 5A). Importantly, 14 days of continuous AF treatment did not result in changes in weight, body conditioning or general activity level in any mouse (data not shown). CBC data on day 14 following AF therapy (**Table 1**) demonstrated no significant toxicity of the drug. At euthanasia, bone marrow sub-population analyses demonstrated no change of HSCs or HSPCs portions in the AF treatment group as compared to the control group (P > 0.05) (Figures 5B), and no significant change of LT-HSC or ST-HSC population (Data not shown), suggesting the bone marrow sustained the stem cell pool by self-renewal and the potential to differentiate to multipotent progenitors as well as HSPCs. Combining with the unchanged effector cells population by CBC data, it demonstrated that AF treatment didn’t cause hematotoxicity. Lastly, at euthanasia common markers of normal tissue injury damage for both the kidney (total serum protein, blood urea nitrogen and creatine) and liver (alkaline phosphatase, alanine transaminase and bilirubin) in the plasma of mice, demonstrated no significant differences with AF treatment (Figure 5C). These results suggest that this dosing regimen is nontoxic to kidneys, liver, and bone marrow as well as demonstrating a trend toward a survival advantage in tumor bearing animals.

## Conclusion

In non-malignant cells, redox metabolism is tightly coupled via non-equilibrium steady-state fluxes of reactive metabolic by-products and electron carriers through redox sensitive signal transduction pathways. It is increasingly recognized that dysregulation of mitochondrial redox metabolism in cancer cells leads to the increased steady-state levels of reactive species including H_2_O_2_ and organic hydroperoxides.^19^ Ionizing radiation leads to the formation of free radicals from the radiolysis of H_2_O to form hydroxyl radical. We are investigating repurposing FDA approved redox active drugs that selectively disrupt the oxidative balance in cancer cells as potential radio-chemo-sensitizers to take advantage of the known dysregulation of oxidative metabolism in cancer cells to improve therapy outcomes.^15,20,21^ In this paper the efficacy of AF treatment in a preclinical xenograft mouse model of SCLC was characterized for future studies as a radio-chemo-sensitizer to improve outcomes in this deadly disease.

These studies demonstrated that AF treatment resulted in dose dependent increases in plasma drug concentration that led to significant inhibition in TrxR in DMS273 xenograft tumors after five days. The average level of the gold found in the plasma with 4 mg/kg IP dosing, 18.4 μM in mice is eleven-fold the average concentration found in humans taking the recommended dose of 6 mg/day orally for seven days, 1.6 μM.^22^ The long half-life (35 days) of AF suggests that higher serum and tumor drug levels can be safely achieved with higher daily dosing and/or continued dosing.^22^ In fact, the primary objective of an ongoing Clinical trial (NCT01737502) is to establish the maximum tolerated dose of AF in combination with sirolimus in lung cancer, including SCLC, with an estimated completion in August, 2023.

AF dosing of 14 days of 4 mg/kg daily IP in female nude mice demonstrated a highly significant 75% reduction in TrxR that was independent of tumor size indicating the AF could penetrate the tumor efficiently. This dose was also well tolerated as demonstrated by general health of the mice, no weight loss, no effect on bone marrow progenitor cells or changes in CBC. In addition, plasma chemistry indicated that there was no evidence of damage to the kidneys or liver. The 14-day AF dosing schedule did demonstrate a trend toward an increase in median overall survival that did not reach statistical significance (p=0.058). Overall, since thoracic irradiation combined with chemotherapy continues to be a standard of care for limited stage SCLC, and is known to induced metabolic oxidative stress in cancer cells, the current data continues to support the hypothesis that AF-induced inhibition of TrxR could represent a promising redox active adjuvant for selective tumor radio-chemo-sensitization.
